# To Signal or Not to Signal? A Non-cooperative Game-Theoretic Approach to Discretionary Communication Between Road Users

**DOI:** 10.1007/s42979-025-04533-w

**Published:** 2025-12-17

**Authors:** Isam Bitar, Albert Solernou Crusat, Richard Romano, David Watling

**Affiliations:** Institute for Transport Studies, University of Leeds, 34-40 University Rd, Leeds, LS2 9JT UK

**Keywords:** Game theory, Communication, Cheap talk, Non-cooperative games, Bayesian games, Emergent cooperation, Discretionary communication, First mover advantage

## Abstract

Reciprocal communication between road users is a vital element of road user interaction. Non-cooperative game theory is an effective framework for modelling and characterising communicative behaviour between road users, which enables the study of emergent benefits for both the issuer and recipient of communicative signals. In this paper, we introduce discretionary communication to gain an advantage over the other road user by masking one’s intent if beneficial to do so. We conduct a series of experiments with simulated interactions and compare interaction outcomes where communication is mandatory against those where communication is discretionary. Our findings further support the premise that non-cooperative game theory is an effective paradigm for modelling and producing emergent behaviours which benefit the network. Moreover, we see that including a layer of *discretionary* communication reaps benefits in interaction outcome to the communicator. It also provides benefits in safety to all parties involved above and beyond the benefits seen from mandatory communication.

## Introduction

The reciprocal interaction between road users in which they engage in competitive, cooperative, and communicative behaviours to negotiate priority and road space is an integral part of navigating the road network. Properly understanding and modelling these interactions is a growing field, especially as autonomous vehicles get closer to technological and market maturity.

Until recently, research on modelling communication as an active component of road user interaction rejected the game-theoretic approach due in part to its perception as a framework that does not lend itself to communicative behaviour. For example, the researchers in [1] relied on an underlying assumption of the existence of a *shared goal* between interacting players to model communicative behaviour. However, we have provided a proof of concept in [2] that non-cooperative game theory (where players only seek to maximise one’s own utility) can indeed provide a robust framework for modelling road user communication descriptively and prescriptively, without the need for an underlying shared goal.

In our recent paper [2], we concluded that issuing and receiving communication to influence interaction outcomes from a non-cooperative game-theoretic perspective could feasibly occur, and that it could produce emergent, population-wide benefits.

The current paper seeks to expand on that paradigm by exploring whether *discretionary* communication further improves a road user’s utility in an interaction. The original model confines communicative behaviour to the Main-Lane Vehicle $M$ to communicate its intent to the Joining Vehicle $J$. It is implied that Vehicle $J$ makes its intent clear to Vehicle $M$ prior to the interaction, hence Vehicle $M$ is always the vehicle to *move first*. The enhanced model we propose in this paper allows Vehicle $J$ to make a *discretionary* choice between the behaviour implied in the original model (signalling the desire to join ahead) or to forego a signal in favour of a ‘surprise manoeuvre’ to attempt to force a lane change.

Thus, we aim to explore the validity of the following two hypotheses as part of this study.

- Vehicles which engage in *discretionary* communication have an advantage (better payoff) over vehicles in the same situation which *always* communicate their intent to their opponents
- Communication in a non-cooperative game-theoretic framework can make interactions safer (fewer crashes and dangerous interactions) and more efficient (better payoffs for all parties involved), even when this communication is optional

One way in which we can measure interaction efficiency is by studying the occurrence of non-ideal outcomes. Non-ideal outcomes are an important metric to measure in the context of road user communication, since they reflect either miscommunication or misreading of one or both road users in an interaction. Such outcomes are non-ideal because they often result in an action by one vehicle that is opposite to what the other intended. For example, at a T-junction, the main-road vehicle may choose to yield to the minor-road vehicle, which chooses not to accept right of way anyway. Thus, the major-road vehicle loses time and momentum, and the minor-road vehicle waits unnecessarily. As such, these outcomes often return worse payoffs to one or both vehicles than what would have been achieved if either vehicle took a different decision. Outcomes like these effectively leave some utility ‘on the table’, and are commonly referred to in game theory as *Pareto inefficient* [3]. We posit that access to better information (via communication) should incrementally reduce the occurrence of non-ideal outcomes. This concept is known as *Pareto improvement*.

To this end, we build on the experimental design we’ve developed in [2], where we simulate the interaction between vehicles in a non-cooperative game-theoretic setting. We develop one set of simulations which forces the agent vehicle into communicating its intent every time, and another which allows it to choose whether to communicate, based on its assessment of the benefit of doing so. By comparing the results, we can gauge the effect of discretionary communication on both the issuer and the recipient of communication, as well as the effect of this behaviour on the safety and efficiency of the interaction in general.

## Literature Review

Historically, the domain of road user interaction has been left as an accessory to microsimulation models of different multi-agent driving scenarios, such as car following [4, 5] and lane changing [6]. In these models, road user interaction is often restricted to collision avoidance. Increasingly, game-theoretic models have emerged to take a more in-depth look at the interaction element itself, especially from an autonomous vehicle’s perspective [7, 8].

Lane-change modelling is a topic which has been explored in depth in the literature. Traditional lane changing models use preset rules to determine the necessity and feasibility of lane changing, irrespective of individual road users’ preferences or constraints [9, 10]. The general formula is that an incentive criterion competes with a safety criterion to dictate whether a lane change occurs. Incentive is often some form of speed or space advantage, whilst safety concerns the risk of collision. Such models at first assumed homogeneity amongst road users, hence the use of a global set of rules. Later models introduced some individuality to the lane change interaction [11–13]. For example, [12] introduced a ‘politeness’ factor which considers the disutility to the rest of the traffic population should the agent carry out a lane change. Conversely, [13] introduced an ‘aggressiveness’ factor which influences an agent’s preference for space over safety. Neither model, however, attempted to build a framework in which an agent advertises these preferences to other road users.

Increasingly, the premise of interaction between two or more agents has become the domain of game theory. One of the first to introduce a game-theoretic lane changing model is Kita [14]. Kita employed a simple, two-player non-cooperative game with complete information, where each player chooses from a set of two strategies, validated and calibrated against real-world data.

Later models extended the game-theoretic approach in several directions. Some built on Kita’s original framework by incorporating variation in vehicle kinematics and more robust payoff functions [15]. Others introduced hierarchical structures to capture interaction at multiple levels. For example, [16] separated long-horizon strategic reasoning from short-horizon tactical games, whilst [17] modelled bounded rationality through “level-k” reasoning, where agent sophistication in anticipating the opponent is layered recursively with every next level.

Another common approach is the adoption of repeated games. Repeated games allow behaviour to unfold across multiple stages, enabling history-dependent strategies such as reciprocity [18]. Kang and Rakha [19] followed this approach to capture ongoing tactical adjustment, whereas [20] applied a receding-horizon method where the game is rebuilt at each timestep without persistent memory or cumulative payoffs. The former enabled the emergence of reciprocal behaviours, whilst the latter emphasised interaction safety.

A more seldom explored but promising paradigm is evolutionary game theory. Iwamura and Tanimoto [21] combined evolutionary game theory with a cellular automaton to demonstrate how emergent stable strategies vary under varying traffic conditions. Bitar et al. [22] extended this line by analysing how spatial factors such as cluster size and vehicle range shape the evolution and success of emergent strategies.

Finally, extensive-form Bayesian games have gained traction as a means of capturing bounded rationality [23]. By allowing agents to update their beliefs about their opponents’ states and preferences, these models move closer to real-world conditions. Applications include [24] on mandatory versus discretionary lane changes, [25] on connected versus non-connected environments, and [2], where we introduced communication itself, through implicit and explicit signals, as an active component of interaction in a game-theoretic setting.

Thus, [2] adds road user interaction to the growing body of fields in which non-cooperative game theory is used to describe and explain emergent cooperative and communicative behaviour [18, 26–34]. This concept carries its own implications regarding interaction with autonomous vehicles, given the general tendency for humans to behave less cooperatively towards machines [35–37]. This means that there is a question to be raised on whether autonomous vehicles should *consider* if it is beneficial to advertise their intent to other road users. Indeed, evidence suggests that communication can be used to deceive other players when there is an asymmetry in available information [34]. We have previously shown that autonomous vehicles would need to perform *better* than human-driven vehicles in terms of interaction outcomes to survive in a mixed population [22, 38]. Therefore, exploring this aspect in the context of road user communication may be of use, especially in the broader context of autonomous vehicle interaction with human road users. This paper builds on that conclusion by looking at the operational/tactical level behaviour, such as discretionary signalling.

Research suggests that most instances of communication between road users are implicit [39–41]. However, explicit communication, less common as it may be, remains an emphatic conveyor of information and road user intent [42]. In our recent work [2], we conceptualised a non-cooperative game as a lane-change scenario in which a Joining Vehicle $J$ desires to change lanes ahead of a Main-Lane Vehicle $M$. The paper concluded that both vehicles benefit from Vehicle $M$ issuing such helpful communication. It is important to note that neither vehicle earned nor lost payoff directly from this communication. That is, the communication did not have an intrinsic utilitarian value. In game theory, this form of communication is known as *cheap talk*, where providing and receiving information is free [43]. The paper’s model also assumed that the vehicles received and interpreted communication perfectly. In the real world, communication may be obscured, ignored, or misunderstood. In fact, Bayesian game-theoretic models exist that are entirely dedicated to the utterance, receipt and understanding of communication between players [44, 45]. Though such a paradigm would add interesting complexity to an interaction model, it was beyond the scope of that study. The paper also assumed that the Joining Vehicle $J$ would implicitly but *unambiguously* make its intent clear that it wishes to join. As such, the question of whether there’s merit to either vehicle to *mask* its intent from the other remains open.

Masking one’s intent may be considered a form of *deception*. Deception is a game-theoretic concept in which the deceiving player limits, distorts, or alters information about the game (usually one’s own attributes, preferences, actions or payoffs) to *trick* the opponent into taking action that favours the deceiver at the expense of the deceived [46, 47]. The topic of deception has been explored in various applications, including sociology [48], politics [49], animal behaviour [50, 51] and even cyber security [52, 53]. By masking its intent, the Joining Vehicle $J$ robs the Main-Lane Vehicle $M$ of the ability to anticipate (and potentially block) Vehicle $J$’s join attempt. To our knowledge, this concept is yet to be explored in the context of road user interaction.

We believe there is merit in investigating the effect of *deception* in road user communication in a non-cooperative game-theoretic setting. To date, research on the topic of road user communication has not explored this aspect. There is a particular interest in studying this concept in the context of the interaction between autonomous vehicles and other road users. Research shows that human road users are likely to behave less cooperatively towards autonomous vehicles. Perhaps, then, masking its intent may prove a useful tool in the autonomous vehicle’s toolbelt, which would help it navigate a potentially unfriendly environment. We aim to explore the feasibility of this form of behaviour in a non-cooperative game-theoretic setting, whether such behaviour would bring benefit to the agent vehicle, and what impact such behaviour may have on general interaction safety and efficiency.

## Method

We conceptualise a discretionary lane change scenario between a Joining Vehicle $J$ and a Main-Lane Vehicle $M$. In this section, we describe an expanded model which we built on top of the model we developed in [2]. In the original model, Vehicle $M$ moves first to allow or block $J$, followed by Vehicle $J$’s response. In the present paper, this basic model is expanded to accommodate an additional, pre-emptive move by Vehicle $J$, where $J$ may decide to *signal* its intent to join ahead or forego a signal to try to *force* a join instead.

We discuss in this section the game-theoretic interaction model, the kinematic framework used to play an interaction, the payoff functions, and the communication element, which is the focus of this paper. We then detail the experimental design adopted in this paper to study the hypotheses we outlined in the introduction.

**Fig. 1:**
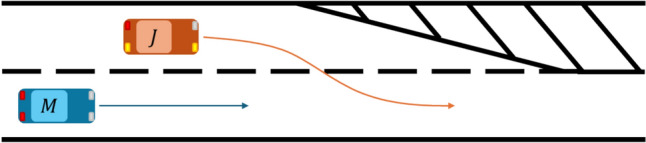
Conceptual layout of the interaction between main-lane vehicle $M$ and joining vehicle $J$

We employ a simple ‘lane change’ operation based on the bi-directional General Motors Car Following Model [54]. Figure 1 illustrates the conceptual layout of the proposed scenario. We use the General Motors model for its relative simplicity and reliance on Newtonian kinematics. This allows us to easily identify and isolate individual parameters to aid in interaction development.

### The Game-Theoretic Interaction Model

**Fig. 2:**
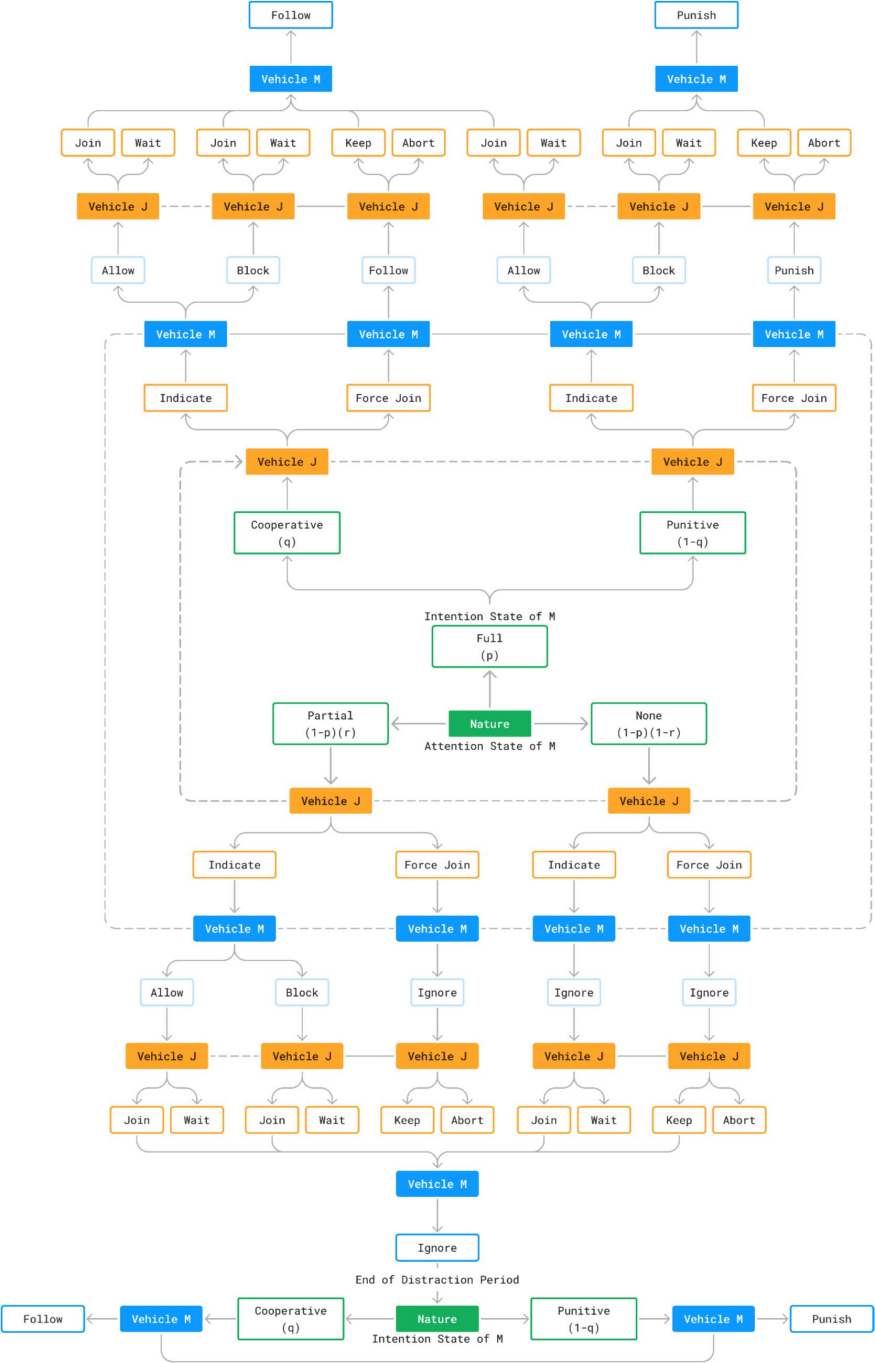
The sequential game between the main-lane vehicle $M$ and the Joining Vehicle $J$ (game tree)

The interaction model employed is a two-player, sequential, non-cooperative Bayesian game between Vehicle $M$ and Vehicle $J$. The extensive form of the model (game tree) is illustrated in Fig. 2. Vehicle $M$ has two stochastic properties whose values are based on predetermined probabilities. These two properties are $\mathrm{attention}$ (possible values: $\mathrm{full/partial/none}$) and $\mathrm{intention}$ (possible values: $\mathrm{cooperative/punitive}$) . $\mathrm{attention}$ relates to Vehicle $M$’s awareness of and responsiveness to Vehicle $J$’s movement. Literature on attention in the context of game-theoretic interaction is scarce [4]. For example, [55] extends the Gipps car following model to incorporate differences in reaction time but falls short of modelling distraction. $\mathrm{intention}$ relates to whether Vehicle $M$ would attempt to punish a lane-change it did not agree to. This is a common concept in game-theoretic interaction models and manifests in different forms [6, 8]. Vehicle $M$ knows whether it is $\mathrm{cooperative}$ or $\mathrm{punitive}$ (solid horizontal line between the branches) but does not know its $\mathrm{attention}$ level (dashed line between the branches). In game-theoretic sequential models, such properties are modelled as *moves by Nature.* $\mathrm{Nature}$ is a third-party entity which selects the values of the stochastic elements of a sequential game based on predetermined probabilities. These elements create uncertainty about the information available to one or more players, which warrant the establishment of *beliefs.* Beliefs are probabilistic assumptions about one or more properties relevant to a player or the interaction. We elaborate further on this concept later in this section.

Vehicle $J$ begins the interaction by choosing whether to $\mathrm{indicate}$ its intent to Vehicle $M$ (e.g., turn signal) or attempt to $\mathrm{force}$ the join without warning. If Vehicle $J$ chooses to $\mathrm{indicate}$, Vehicle $M$ may choose to $\mathrm{allow}$ Vehicle $J$ to join or $\mathrm{block}$ its attempt. However, Vehicle $M$ does not get the opportunity to do so if the interaction is forced, or if $M$’s $\mathrm{attention}$ state is $\mathrm{none}$ (note the absence of this step from the respective branches of the game tree). Once $M$ has made its first decision (if applicable), Vehicle $J$ chooses whether to $\mathrm{join}$ ahead of $M$ or $\mathrm{wait}$ until $M$ has passed. If $J$ had previously attempted to $\mathrm{force}$ the join, it may at this stage choose to $\mathrm{keep}$ its original decision and continue, or $\mathrm{abort}$ the manoeuvre and wait instead. Finally, Vehicle $M$ must take a further action if Vehicle $J$ chooses to $\mathrm{join}$ or $\mathrm{keep}$. This final action depends on Vehicle $M$’s $\mathrm{attention}$ and $\mathrm{intention}$ states. If $M$’s $\mathrm{attention}$ state is $\mathrm{full}$, it will adopt a $\mathrm{follow}$ trajectory when $\mathrm{cooperative}$ and a $\mathrm{punish}$ trajectory when $\mathrm{punitive}$. $\mathrm{punishment}$ entails tailgating Vehicle $J$ to induce a negative headway utility (see $U_{h}$ in the Payoffs section). If Vehicle $M$’s $\mathrm{attention}$ state is $\mathrm{partial}$ or $\mathrm{none}$, it will $\mathrm{ignore}$ Vehicle $J$ and continue moving as if it were in free flow and will not take measures to prevent a collision if one were imminent. This state only lasts for a finite amount of time, after which Vehicle $M$ employs $\mathrm{follow}$ or $\mathrm{punish}$ as appropriate.    

### The Kinematic Model

The movement model is a discretised approach, where at each timestep each vehicle’s kinematic properties are evaluated. Acceleration is governed by a modified version of the bi-directional General Motors Car Following Model [2, 54] subject to the relevant acceleration and deceleration constraints for each vehicle. The general formula is illustrated in Eq. (1). All other kinematic properties are governed by Newtonian equations of motion.

1 $$a_{x,n + 1} = { }\frac{{ \alpha_{x} { }\left( {v_{x,n} } \right)^{m} }}{{(\Delta s_{n})^{l} }}{ }\Delta v_{n} $$

where $a_{x,n + 1}$ is the acceleration of Vehicle $x$ at the start of the next timestep $n + 1$. $v_{x,n}$ is the velocity of Vehicle $x$ at the current timestep $n$. $\Delta s_{n}$ is the distance between Vehicle $x$ and its car-following target at timestep $n$. $\Delta v_{n}$ is the velocity difference between Vehicle $x$ and its target at timestep $n$. $\alpha_{x}$ is a sensitivity factor which governs Vehicle $x$’s acceleration rate to maintain car-following behaviour. Higher $\alpha_{x}$ means more conservative movement. $m, l$ are parametric factors which in this study are set to 1.    

$a_{x,n + 1}$ is constrained by the agent vehicle’s acceleration preferences and physical limitations (see Table 2 for the value ranges used in this paper).

Vehicle $M$’s $\mathrm{block}$ movement is governed by Eq. (2), derived from Newtonian equations of motion. It is the only acceleration value which is not governed by Eq. (1).

2 $$a_{M,n + 1} = a_{J,\max} + 2\,\frac{{\Delta s_{n} + \left( {v_{J,n} - v_{M,n} } \right)t_{\text{LC}} }}{{t_\mathrm{LC}}^{\,2}} $$

where $a_{Mn + 1}$ is the acceleration of Vehicle $M$ at the start of the next timestep $n + 1$. $a_{J,\max}$ is Vehicle $J$’s maximum acceleration. $\Delta s_{n}$ is the distance between Vehicle $J$ and Vehicle $M$ at timestep $n$. $v_{J,n}$, $v_{M,n}$ are Vehicle $J$ and Vehicle $M$’s velocities, respectively, at timestep $n$. $t_\mathrm{LC}$ is the lane change duration.

A brief description of the model’s timesteps is outlined below.

- $T_{0}$ = 0; begins the interaction the moment Vehicle $J$ takes its first action
- $T_{1}$ = Vehicle $M$’s Decision-Reaction Time $D_{M}$; Vehicle $M$ reacts to Vehicle $J$’s initial action ($\mathrm{allow/block}$ if $\mathrm{indicate}$, $\mathrm{follow/punish/ignore}$ if $\mathrm{force}$)    
- $T_{2}$ = Vehicle $J$’s Decision-Reaction Time $D_{J}$; Vehicle $J$ takes its second decision ($\mathrm{join/wait}$ if first decision was $\mathrm{indicate}$, $\mathrm{keep/abort}$ if first decision was $\mathrm{force}$)
- $T_{3}$ = $D_{M}$; Vehicle $M$ reacts to Vehicle $J$’s second decision ($\mathrm{follow/punish/ignore}$)
- $T_{4} - T_{n - 1}$ = 0.5 seconds; regular timesteps where the vehicles employ the modified General Motors Car Following Model as the interaction resolves
- $T_{n}$; the final timestep in an interaction, which is reached if a crash is detected, a predefined maximum duration is reached, or when all of the following interaction conclusion conditions [2] are met:
  - $$\left| {a_{M,n} } \right| \le 0.01{ }\,\mathrm{m/s^{2}}$$  
  - $$\left| {a_{J,n} } \right| \le 0.01{ }\, \mathrm{m/s^{2}}$$  
  - If Vehicle $J$’s second action is $\mathrm{wait}$ or $\mathrm{abort}$, Vehicle $J$’s current time headway $HW_{J,n}$ is greater than or equal to Vehicle $J$’s minimum acceptable time headway $HW_{J,\min}$  

### Payoff Functions

The payoff for each vehicle is a function of several components. These are listed and described below.

### Ride Comfort ${\varvec{U}}_{{\varvec{a}}}$

Ride comfort is best represented as the change in acceleration over time (jerk). Acceleration is a common element in game payoffs in the literature [13, 14, 24]. In this model, we base ride comfort on both the acceleration values at each timestep (with respect to the vehicle’s comfortable acceleration value $a_{c}$) and a measure of jerk over the interaction period (measured as the standard deviation of acceleration about its mean). The jerk element of the payoff calculation allows for a utilitarian distinction between different strategies. For example, a failed $\mathrm{block}$ attempt where Vehicle $M$ accelerates then sharply decelerates will yield a worse payoff than a sustained sharp deceleration of equal magnitude.

Ride comfort $U_{a}$ applies to all interaction possibilities, and it is calculated as prescribed in Eq. (3).

3 $$ U_{a} = { }\frac{1}{T}\mathop \sum \limits_{n = 1}^{T} \min \left( {\left( {1 - \frac{{a_{n} }}{{a_{c} { } \vee { }d_{c} }}} \right) \times t_{n} { },{ }0} \right){ } - { }\sqrt {\frac{1}{T}\mathop \sum \limits_{n = 1}^{T} \left( {a_{n} - \overline{a}} \right)^{2} } $$

where $T$ is the total count of the interaction’s timesteps. $a_{n}$ is the agent vehicle’s acceleration at timestep $n$. $\overline{a}$ is the mean of $\{ a_{1} , a_{2} , ..., a_{T} \}$. $a_{c}$, $d_{c}$ are the vehicle’s maximum comfortable acceleration and deceleration, respectively. $a_{c}$ is used in the denominator of the first term if $a_{n} \ge 0$, else $d_{c}$. $t_{n}$ is the duration of timestep $n$.

### Time Headway ${\varvec{U}}_{{\varvec{h}}}$

Time headway is another common metric in many interaction models and, together with time to collision (TTC), forms the basis of interaction safety [14, 56]. In this study, we define $U_{h}$ as a function of the minimum time headway achieved during the interaction with respect to the agent vehicle’s minimum acceptable headway $HW_{\min}$. Time headway $U_{h}$ applies to all interactions where Vehicle $J$ chooses or attempts to join at any point. It is calculated as prescribed in Eq. (4).

4 $$ U_{h} = \left\{ {\begin{array}{*{20}l} {U_{crash} } \hfill & {if \exists HW_{n \in T} \le 0} \hfill \\ {\mathop {\min }\limits_{n \in T} \left( {1 - \frac{{HW_{min} }}{{HW_{n} }}} \right)} \hfill & {otherwise} \hfill \\ \end{array} } \right. $$

where $n$, $T$ are the current timestep and the set of all timesteps, respectively. $U_\mathrm{crash}$ is the disutility from being involved in a crash. $HW_{n}$ is the vehicle’s time headway with respect to the lead vehicle at timestep $n$. $HW_{\min}$ is the vehicle’s minimum acceptable time headway.        

### Speed Difference ${\varvec{U}}_{{\varvec{v}}}$

Speed difference concerns the steady states before and after an interaction. It is often used as part of the incentive function which triggers a lane change [15] or as part of the reward for changing lanes [24, 57].

For Vehicle $M$, the speed difference payoff is based on the difference between the vehicle’s initial and final (stable) velocities, where a lower final velocity (caused by following a slower $J$) brings a negative utility to $M$. Similarly, if Vehicle $J$ opts to $\mathrm{wait}$, $U_{v}$ becomes the difference between $J$’s desired velocity after the lane change and $M$’s stable velocity. This would mean that $J$ would incur a penalty if it is forced to join *behind* a Vehicle $M$ that is slower than Vehicle $J$’s target (desired) velocity. Speed difference $U_{v}$ is applicable to either vehicle when it is the *lag* vehicle. This is in a variation to the original application of $U_{v}$ in [2], where it also applied to Vehicle $J$ as a lead vehicle if at any point during the interaction it was forced to accelerate beyond its initial or desired velocities. We have opted to remove Vehicle $J$’s payoff function when it is the lead vehicle from the current study, since Vehicle $M$’s highest achieved velocity did not factor into its own $U_{v}$, and so it did not seem to make sense that we consider this element for Vehicle $J$ but not for Vehicle $M$. Thus, $U_{v}$ is calculated according to Eqs. (5) and (6) below.

5 $$U_{v,M} = { }1 - \frac{{v_{M,0} }}{{\min \left( {v_{M,0} ,v_{J,D} } \right)}} $$

6 $$U_{v,J} = { }1 - \frac{{v_{J,D} }}{{\min \left( {v_{M,0} ,v_{J,D} } \right)}} $$

where $v_{M,0}$ is Vehicle $M$’s initial (and stable) velocity, $v_{J,D}$ is Vehicle $J$’s desired velocity after the lane change.

### Time Penalty ${\varvec{U}}_{{\varvec{t}}}$

Many game-theoretic models consider some form of time penalty in their payoffs, typically represented as time spent in the undesirable lane [14, 15, 56]. In the proposed model, Vehicle $J$ is subject to $U_{t}$ when it chooses to $\mathrm{wait}$. It is a function of the amount of time $J$ needs to wait for $M$ to pass before it can $\mathrm{join}$ behind it. It is calculated as the time until $HW_{J,n}$ is equal to or greater than $J$’s minimum acceptable time headway $HW_{J,\min}$. If an interaction ends before this condition is met, the remainder is estimated based on Vehicle $M$’s velocity and position at the end of the interaction relative to Vehicle $J$. The payoff value is then multiplied by a factor $\mu$ that is specific to that instance of $J$. $\mu$ represents Vehicle $J$’s *sensitivity* to losing time. The higher, the less tolerant $J$ is to waiting its turn and the more likely it is to make riskier merges to avoid the wait. The calculation is mathematically represented in Eq. (7).

7 $$ \begin{aligned} t_{sum} & = \mathop \sum \limits_{n = 1}^{T} HW_{Jn} \left[ {HW_{Jn} < HW_{Jmin} } \right] \\ t_{extr} & = \left( {\frac{{HW_{Jmin} v_{J0} + s_{JT} - s_{MT} }}{{0.5\left( {v_{M0} + \max \left( {v_{M0} ,v_{MT} } \right)} \right) - v_{J0} }}} \right) \\ U_{tJ} & = \left\{ {\begin{array}{*{20}l} {U_{crash}^{*} } \hfill & {if{ }v_{J0} \ge v_{M0} } \hfill \\ { - \mu \times t_{sum} } \hfill & {if HW_{JT} > HW_{Jmin} } \hfill \\ { - \mu \left( {t_{sum} + t_{extr} } \right)} \hfill & {otherwise} \hfill \\ \end{array} } \right.{ } \\ \end{aligned} $$

*$U_\mathrm{crash}$ is used here to prevent Vehicle $J$ from waiting indefinitely for a slower $M$ to pass

where $T$ is the total count of the interaction’s timesteps. $HW_{J,n}$, $HW_{J,T}$ are Vehicle $J$’s time headway with respect to Vehicle $M$ at timestep $n$ and the final interaction timestep, respectively. $HW_{J,\min}$ is Vehicle $J$’s minimum acceptable time headway. $v_{J,0}$, $v_{M,0}$ are Vehicle $J$’s and Vehicle $M$’s initial velocities, respectively. $s_{J,T}$, $s_{M,T}$ are Vehicle $J$’s and Vehicle $M$’s final positions, respectively. $v_{M,T}$ is Vehicle $M$’s final velocity. $\mu$ is a sensitivity factor which represents Vehicle $J$’s tolerance to losing time.        

### Decision Making

The goal for each vehicle is rooted in fundamental non-cooperative game theory: to maximise one’s own payoff. The structure of the payoff functions ensures that the best interaction is no interaction (maximum possible payoff is zero). This would prevent vehicles from seeking conflict when one is unnecessary. Each vehicle will *simulate* an interaction with its opponent to establish expected payoffs using assumptions made about the opponent’s attributes, which are discussed later in the experimental design section. For Vehicle $M$, this is done for both the $\mathrm{block}$ and $\mathrm{allow}$ actions, then backward induction is used to determine the best action. For Vehicle $J$, the expected payoffs for each of its actions are calculated for all $M$’s possible $\mathrm{attention}$ and $\mathrm{intention}$ combinations. Each is then multiplied by the appropriate probability value to produce the total expected payoff per action.

Table 1 provides a summary of the applicable payoff components depending on the action(s) taken.

**Table 1:** Payoff composition for each vehicle given the appropriate action set

Action set(s)	Vehicle *M*	Vehicle *J*
$M: \text{any action}$ $J: \mathrm{join/keep}$	$U_{a} + U_{h} + U_{v}$	$$\begin{aligned} & pq\left( {U_{{a_{pq} }} + U_{{h_{pq} }} } \right) + p\left( {1 - q} \right)\left( {U_{{a_{{p\left( {1 - q} \right)}} }} + U_{{h_{{p\left( {1 - q} \right)}} }} } \right) \\ & + \left( {1 - p} \right)q\left( {U_{{a_{{\left( {1 - p} \right)q}} }} + U_{{h_{{\left( {1 - p} \right)q}} }} } \right) \\ & + \left( {1 - p} \right)\left( {1 - q} \right)\left( {U_{{a_{{\left( {1 - p} \right)\left( {1 - q} \right)}} }} + U_{{h_{{\left( {1 - p} \right)\left( {1 - q} \right)}} }} } \right) \\ \end{aligned}$$
$M: \text{any action}$ $J:\mathrm{wait/abort}$	$U_{a}$	$$\begin{aligned} & pq\left( {U_{{a_{pq} }} + U_{{v_{pq} }} + U_{{t_{pq} }} } \right) + p\left( {1 - q} \right)\left( {U_{{a_{{p\left( {1 - q} \right)}} }} + U_{{v_{{p\left( {1 - q} \right)}} }} + U_{{t_{{p\left( {1 - q} \right)}} }} } \right) \\ & + \left( {1 - p} \right)q\left( {U_{{a_{{\left( {1 - p} \right)q}} }} + U_{{v_{{\left( {1 - p} \right)q}} }} + U_{{t_{{\left( {1 - p} \right)q}} }} } \right) \\ & + \left( {1 - p} \right)\left( {1 - q} \right)\left( {U_{{a_{{\left( {1 - p} \right)\left( {1 - q} \right)}} }} + U_{{v_{{\left( {1 - p} \right)\left( {1 - q} \right)}} }} + U_{{t_{{\left( {1 - p} \right)\left( {1 - q} \right)}} }} } \right) \\ \end{aligned}$$

$p$, $q$ are the probability that $M$’s $\mathrm{attention}$ state is $\mathrm{full}$ and $\mathrm{intention}$ is $\mathrm{cooperative}$, respectively

### Communication

Our work in [2] provided a pathway for Vehicle $J$ to update its *prior beliefs* on Vehicle $M$’s stochastic states via receiving useful communication from Vehicle $M$. The main contribution of this paper is to expand on that model by allowing Vehicle $J$ to choose whether to communicate *its* intent to join and explore the impact of this discretionary communication on the interaction outcome. Communication in this study takes place in three distinct stages.

### Stage 0: Initial Communication from Vehicle $M$ to Vehicle $J$

Before Vehicle $J$ begins the interaction, it will observe Vehicle $M$’s current state and process any communication Vehicle $M$ may engage in. We characterise *eye contact* as a form of (explicit) communication that is available at this stage. That is, Vehicle $J$ will perceive eye contact with Vehicle $M$ as an explicit signal that Vehicle $M$ has *seen* Vehicle $J$. We discuss how this is quantified later in the experimental design.

### Stage 1: Discretionary Communication from Vehicle $J$ to Vehicle $M$

At the beginning of the interaction, Vehicle $J$ *has the option* to issue communication to Vehicle $M$ about its intent to join ahead. We have designed the game in a way where Vehicle $J$ is incentivised to force-join where possible. However, if Vehicle $J$ decides against forcing the join, it will $\mathrm{indicate}$ its intent to Vehicle $M$ and await a response. This gives Vehicle $M$ the opportunity to $\mathrm{allow}$ or $\mathrm{block}$ the request.

### Stage 2: Further Communication from Vehicle $M$ to Vehicle $J$

Following Vehicle $J$’s initial decision ($\mathrm{indicate/force}$), Vehicle $M$ will signal its intent explicitly and implicitly to Vehicle $J$. We use the same signalling paradigm in this case as that in [2], which consists of a mixture of implicit signals (acceleration) and explicit signals (e.g., eye contact, a gesture, flashing of headlights). We elaborate further on these signals in the experimental design section.

### Updating Beliefs: Bayesian Inference

Vehicle $M$’s $\mathrm{attention}$ and $\mathrm{intention}$ states are assigned upon the vehicle’s instantiation. We outline the base probabilities used for these states in the experimental design section. The base probabilities are known to Vehicle $J$ as *prior beliefs*. Vehicle $J$ will use the communication it receives from Vehicle $M$ in stages 0 and 2 to incrementally *update* these beliefs in accordance with Bayes’ Theorem [58].

8 $$ P\left( {H{|}E} \right) = \frac{{P\left( H \right) P\left( {E{|}H} \right)}}{P\left( E \right)} $$

where $P\left( {H{|}E} \right)$ is the probability that $H$ is true given Observation $E$ (*posterior belief*), $P\left( H \right)$ is the base probability that $H$ is true within the population (*prior belief*), $P\left( {E{|}H} \right)$ is the probability of observing $E$ given that Property $H$ is true (*likelihood*), $P\left( E \right)$ is the total probability of observing $E$ within the population

In the original model [2], Vehicle $J$ assigned a single Bayesian probability to both $\mathrm{distracted}$ states. In the expanded model, Vehicle $J$ assigns a separate Bayesian probability to Vehicle $M$’s probability to be $\mathrm{distracted} \wedge \mathrm{cooperative}$, or to be $\mathrm{distracted} \wedge \mathrm{punitive}$. We believe this improves the Bayesian inference process in the interaction.

### Experimental Design

To properly investigate the impact of two-way discretionary communication on interaction outcomes, we run a simulation as a $\text{Control Group}$ where neither vehicle engages in any form of explicit communication, nor does either vehicle read any implicit signals from the other. In addition, we run two test groups: $\text{Test Group I}$ and $\text{Test Group II}$, each with a different style of communication.

Each vehicle’s attributes and kinematic conditions are generated from set ranges prior to the interaction itself. These are outlined and described in Table 2. The base probability values assigned to Vehicle $M$’s $\mathrm{attention}$ and $\mathrm{intention}$ states are outlined in Table 3. Table 4 gives a breakdown of the different communicable signals employed in this experiment and how these signals translate to Bayesian likelihoods. The likelihoods are in turn based on the signalling probabilities shown in Table 5.

**Table 2:** Values and value ranges used in the simulation (based on values used in [2])

Property/constant	Description	Value/value range
$a_{c}$	Maximum comfortable acceleration	(0.20, 2.00)$\,\mathrm{m/s^{2}}$
$a_{\max}$	Maximum allowable acceleration	(2.50, 3.50) $\,\mathrm{m/s^{2}}$ [59]
$d_{c}$	Maximum comfortable deceleration	(− 0.50, − 1.50)$\,\mathrm{m/s^{2}}$
$HW_{\min}$	Minimum acceptable time headway	(0.50, 3.50)$\,\mathrm{s}$
$DT$	Decision time	(0.50, 1.50)$\,\mathrm{s}$
$\alpha_{P}$	Punitive sensitivity factor (exclusive to $M$)	(0.15, 0.35)
$v_{0}$	Initial velocity	$M$: (8, 18) $\,\mathrm{m/s}$; $J$: (4, 10)$\,\mathrm{m/s}$
$s_{0}$	Initial distance between the vehicles	(14, 89)$\,\mathrm{m}$
$$v_{D}$$	Desired velocity (exclusive to $$J$$)	(0.75, 1.50) × $$v_{0,J}$$$$\,\mathrm{m/s}$$
$\omega$	$\mathrm{wait}$ penalty reduction factor	(0.10, 0.20)
$\left| {i_\mathrm{distraction} } \right|$	Total number of distraction timesteps	10 (5–8 $\,\mathrm{s}$)
$i_{\max}$	Maximum number of timesteps	63 (31.5–34.5 $\,\mathrm{s}$)
$t_\mathrm{LC}$	Lane change duration	5 [60, 61]
$\alpha,{ }m,{ }l$	General motors sensitivity factors	1
$t_{n}$	Timestep $n$ (from $T_{4}$ onward)	0.5 $\,\mathrm{s}$
$t_\mathrm{f}$	Phantom vehicle time headway *	4 $\,\mathrm{s}$
$U_\mathrm{crash}$	Crash penalty	− 250
$d_{\max}$	Maximum safe deceleration	− 4.5 $\,\mathrm{m/s^{2}}$ [59, 62]

*Used in free-flow mode to simulate gradual return to initial/desired velocity

**Table 3:** The base probability for each stochastic property of $M$ (based on values used in [2])

Property/state	Base probability (*J*'s Prior elief)
$\mathrm{attentive} \left( p \right)$	0.75
$\mathrm{cooperative} \left( q \right)$	0.6
$\text{fully distracted} \left( r \right)$	0.5

**Table 4:** Probabilities of Vehicle $M$ issuing various communicative signals

Signal category	Description	Probability of occurrence			
		Attentive cooperative	Attentive punitive	Distracted cooperative	Distracted punitive
Implicit: acceleration	$$M $$ alters its velocity as appropriate	1	1	0.5	0.5
Explicit: attention e.g. eye contact	$M$ makes eye contact with $J$	0.9	0.9	0.05	0.05
Explicit: intention e.g. gestures	$M$ issues a cooperative signal (if $\text{allow}$)	0.8	0.2	0.1	0.05
	$M$ issues a threatening signal (if $\text{block}$)	0.1	0.8	0.05	0.1

**Table 5:** Breakdown of the likelihoods of each signal given vehicle $M$'s different possible stochastic attributes

Signal category	Value	Description	$P\left( {E|H} \right)$ *			
			Attentive cooperative	Attentive punitive	Distracted cooperative	Distracted punitive
Implicit: accelera-tion	0	$J$ observes no acceleration from $M$	0.05	0.05	0.55	0.55
	1	$J$ observes deceleration from $M$	0.55	0.3	0.25	0.15
	− 1	observes acceleration from	0.4	0.65	0.2	0.3
Explicit: $\mathrm{attention}$ e.g. eye contact	0	$J$ is unable to make eye contact with	0.1	0.1	0.95	0.95
	1	$J$ makes eye contact with	0.9	0.9	0.05	0.05
Explicit: $\mathrm{intention}$ e.g. gestures	0	$J$ does not observe an intention signal from $M$	0.585	0.47	0.9275	0.9225
	1	$J$ observes a positive-intent signal from $M$	0.36	0.09	0.045	0.0225
	− 1	$J$ observes a negative-intent signal from $M$	0.055	0.44	0.0275	0.055

*$P\left( {E{|}H} \right)$ values are based on results obtained from a pilot simulation run of 30,000 interactions

In the $\text{Control Group}$, Vehicle $J$ relies solely on the base probabilities of Vehicle $M$’s $\mathrm{attention}$ and $\mathrm{intention}$ states as *prior beliefs*. Furthermore, Vehicle $J$ does not engage in any action at ${T}_{0}$. The interaction effectively begins at ${T}_{1}$, at Vehicle $M$’s $\mathrm{allow/block}$ decision node.

In $\text{Test Group I}$, Vehicle $J$ always advertises its intent to join and Vehicle $M$ employs the full suite of communication signals described in Table 4 during its first decision phase. Vehicle $J$ can interpret all signals issued implicitly or explicitly by Vehicle $M$. As with the $\text{Control Group}$, the interaction effectively begins at ${T}_{1}$. This group is analogous to [2]’s Test Group B. We include this test group in this experiment to benchmark our expanded model’s results against the original findings of [2], and to provide a second benchmark for the main test group of this paper. All communication in $\text{Test Group I}$ is mandatory and takes place the moment Vehicle $M$ makes its $\mathrm{allow/block}$ decision.

$\text{Test Group II}$ is the main test group of this paper. In this test group, Vehicle $J$ begins the interaction at ${T}_{0}$ by choosing whether to $\mathrm{indicate}$ (signal intent as usual) or $\text{force join}$ without a signal. In this scenario, $\mathrm{indicating}$ is equivalent to $\text{Test Group I}$ and the $\text{Control Group}$ in that it passes on the first decision to Vehicle $M$. Choosing $\text{force join}$ allows Vehicle $J$ to *take control* of the interaction by *moving first*. Communication in $\text{Test Group II}$ follows the stages outlined earlier in this section under Communication.

Each simulation group ($\text{Control Group}$, $\text{Test Group I}$ and $\text{Test Group II}$) comprises ten simulations of 30,000 interactions each. Every interaction involves a unique instance of Vehicle $M$ and Vehicle $J$. Each vehicle is spawned with attributes and preferences generated randomly from a uniform distribution of the preset ranges shown in Table 2. Each of the ten simulations uses a predefined random generator seed, which is repeated in all the three experiment groups. Using ten different random seeds per simulation group ensures that the findings are repeatable across different rolls of the randomiser dice. Reusing each random seed across all three experiment groups ensures that every resultant interaction has a corresponding mirror in the other experiment groups. That is, interactions can be paired and compared using pairwise statistics, such as the paired samples t-test.

The experiment is completed under two different rulesets. We use the same rulesets set out in the original model [2], which are briefly described below.

*Ruleset 1 (Transparent)*: both vehicles have full knowledge of each other’s attributes and preferences, apart from the stochastic elements of $\mathrm{attention}$ and $\mathrm{intention}$. This is a game of near-complete information, where the uncertainty is confined to these two elements and allows for the study of the effect of communication without noise.

*Ruleset 2 (Blind)*: neither vehicle has any knowledge of the other’s attributes and preferences. They assume that their opponent has the same attributes as they do. The only accurate information that’s available is on the other vehicle’s velocity and position. This game of incomplete information allows for the study of communication in a more noisy/uncertain setting.

**Fig. 3:**
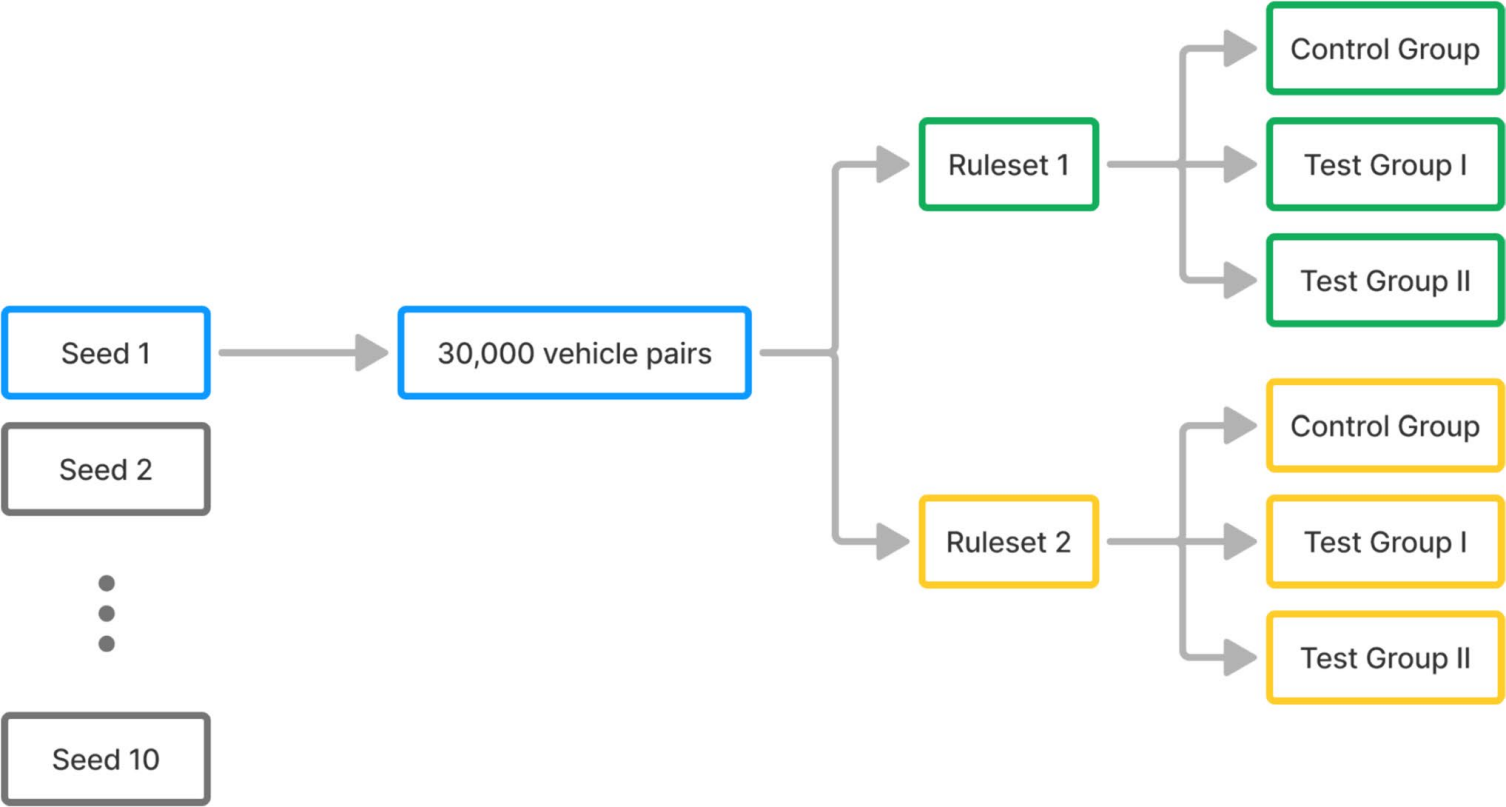
Schematic of the composition of the simulation suite

A visual representation of the simulation suite is shown in Fig. 3.

### Hardware and software requirement

The simulations are conducted in a purpose-built simulation suite developed in Python 3.11.0 by the authors and run on a Windows 11 PC with a 2.9-GHz, six-core processor. Please refer to the Data availability section under Statements and declarations for the source code.

## Results

All simulations were completed successfully, with no aborted or incomplete runs. The results from the simulations are aggregated and presented in Table 6. Overall, the simulations produced interactions where vehicles behaved according to their characteristics, preferences and physical positioning. Vehicles also generally favoured safer interactions and avoided taking catastrophic risks. There were no recorded crashes under Ruleset 1, and the average number of recorded crashes under Ruleset 2 was 113 crashes per 300,000 interactions (0.04%). The distribution of starting conditions was balanced, as most scenarios resulted in a relatively even split between $\mathrm{join}$ and $\mathrm{wait}$ outcomes. (average 55.54% and 44.46%, respectively across both rulesets). Non-ideal outcomes, i.e. $\text{allow/wait}$ and $\mathrm{block/join}$ (where applicable) were low in number, but non-trivial (1.89% and 0.97%, respectively across both rulesets).

**Table 6:** Summary of simulation results

Metric	Ruleset 1 (transparent)			Ruleset 2 (blind)		
	Control	Group I	Group II	Control	Group I	Group II
allow/join*	49.36%	49.76%	63.07%	49.93%	50.39%	64.91%
allow/wait	1.96%	1.57%	1.62%	2.37%	1.91%	1.92%
block/join	1.15%	0.67%	0.00%	2.20%	1.82%	0.00%
block/wait	47.52%	48.00%	35.30%	45.50%	45.89%	33.17%
Near misses	0.44%	0.14%	0.08%	0.63%	0.43%	0.19%
Crashes	0.00%	0.00%	0.00%	0.07%	0.04%	0.01%
Average payoff (vehicle $M$)	− 0.751	− 0.724	− 0.758	− 1.059	− 0.952	− 0.809
One-tailed paired t-test (vs Control)	–	< 0.01	< 0.01	–	< 0.01	< 0.01
One-tailed paired t-test (vs Group I)	–	–	< 0.01	–	–	< 0.01
Average payoff (vehicle $J$)	− 0.511	− 0.492	− 0.403	− 0.761	− 0.654	− 0.435
One-tailed paired t-test (vs Control)	–	< 0.01	< 0.01	–	< 0.01	< 0.01
One-tailed paired t-test (vs Group I)	–	–	< 0.01	–	–	< 0.01

*For test/group II, allow/join also includes forced joins, regardless of vehicle $M$'s $\text{intention}$

### Ruleset 1 (Transparent)

Ruleset 1 produced safer and more efficient interactions than Ruleset 2. 2.63% of all interactions under Ruleset 1 had non-ideal outcomes ($\text{allow/wait}$ or $\mathrm{block/join}$). Ruleset 1 had no crashes and an average near-miss rate (defined as having a time headway of less than half a second at any point during the interaction) of 0.22%.    

Compared to the $\text{Control Group}$, $\text{Test Group I}$ came with a 68.4% decrease in near-misses and a 3.6% and 3.7% improvement of average utility (payoff) for Vehicle $M$ and Vehicle $J$, respectively. These figures are statistically significant ($p$ < 0.01).  

$\text{Test Group II}$ of Ruleset 1 further improves the interaction outcomes, providing an 81.6% decrease in near misses on the $\text{Control Group}$, and a 21% improvement of average utility for Vehicle $J$. Conversely, however, $\text{Test Group II}$ saw a 4.8% *worsening* of average utility for Vehicle $M$ compared to the $\text{Control Group}$. The figures are statistically significant against both the $\text{Control Group}$ and $\text{Test Group I}$ ($p$ < 0.01).        

### Ruleset 2 (Blind)

Ruleset 2 had a higher occurrence of non-ideal outcomes (4.08%) and near misses (0.78%). Unlike Ruleset 1, some interactions under Ruleset 2 resulted in a crash (0.07%). Furthermore, Ruleset 2 showed a markedly worse average utility for both Vehicle $M$ and Vehicle $J$ compared to Ruleset 1 (41.06% and 48.93% worse, respectively).

$\text{Test Group I}$’s incidence of near misses saw a 31% reduction in near misses compared to the $\text{Control Group}$. Similarly, $\text{Test Group I}$’s crash rate went down by 41%. The average utility improvement compared to the $\text{Control Group}$ for Vehicle $M$ and Vehicle $J$ was 10% and 14%, respectively. The improvements are statistically significant ($p$ < 0.01).

$\text{Test Group II}$ continued this trend by further improving interaction safety and efficiency across the board. Thus, $\text{Test Group II}$ saw crashes and near misses reduced by 87% and 70%, respectively, compared to the $\text{Control Group}$. As for average utility, an improvement of 43% compared to the $\text{Control Group}$ is seen for Vehicle $J$. Unlike in Ruleset 1, Ruleset 2’s $\text{Test Group II}$ also improved Vehicle $M$’s average utility by 15% compared to the $\text{Control Group}$.    

## Discussion

We draw a comparison between our results and the results presented in the original paper [2]. We also compare between the different rulesets and simulation groups which form the experimental design of this paper, discuss the different patterns and trends that emerge and pit the results against our hypotheses to draw conclusions.

### Comparison with the Results of the Original Paper [2]

Comparing $\text{Control Group}$ and $\text{Test Group I}$ to their equivalent in [2], this work produces consistently similar results. The $\text{Control Group}$ of both rulesets exhibited interaction outcomes that are on par with what is seen in the Control Groups of the original paper [2], if not slightly safer. The marginal increase in safety may be attributable to the addition of the extra interaction timestep described at the beginning of the Methods section, which allows more time for vehicles to react to one another. Similarly, $\text{Test Group I}$ under both rulesets corroborate the general trends seen in [2] in the equivalent Test Groups B.    

Both Rulesets produce progressively safer and more efficient interactions with better communication. Ruleset 2 returns the largest percent improvement in safety across the board compared to Ruleset 1. These findings corroborate and further reinforce the findings in [2]. Namely that communication improves interaction safety and vehicle payoffs in a statistically significant manner, and that the effect is more pronounced when information is more limited (as in Ruleset 2). We explore some of these findings in more detail below.

### Effect on Interaction Advantage (Payoffs)

The main exception to the trend of improvement with communication is Vehicle $M$’s average payoff under Ruleset 1. $\text{Test Group II}$ saw a statistically significant *worsening* compared to both the $\text{Control Group}$ and $\text{Test Group I}$. This suggests that Vehicle $M$ finds itself at a relative disadvantage when $J$ has the option to force a join, compared to the scenarios where it does not. This is especially evident when there are few other benefits to be gained (there were no crashes in any of the test groups under Ruleset 1, the reduction of which would have helped offset this disadvantage). Yet, the total interaction payoff (payoff of $M$ + payoff of $J$) is higher in $\text{Test Group II}$ than $\text{Test Group I}$ and $\text{Control Group}$. This suggests that the interaction overall is more effective. Interestingly, however, Vehicle $M$ sees a significant *improvement* in its own payoff in $\text{Test Group II}$ compared to $\text{Test Group I}$ and $\text{Control Group}$ under Ruleset 2. The reduction in other utility-damaging factors such as crashes raises Vehicle $M$’s average payoff into a net improvement. This is an important result, since it shows that even when Vehicle $J$ engages in seemingly aggressive behaviour, benefits could be had for both parties in the interaction.        

In contrast, Vehicle $J$’s average payoff significantly improved under both Rulesets in $\text{Test Group II}$ compared to $\text{Test Group I}$ and $\text{Control Group}$. This suggests that Vehicle $J$ can see an advantage from masking its intent and bullying its way into the merge. More broadly, Vehicle $J$’s clear advantage over Vehicle $M$ under both Rulesets’ $\text{Test Group II}$ is a testament to what is known as the *first mover advantage.* In game theory, the *first mover advantage* is the advantage a player gains by being the first to carry out an action in a sequential game. An example of this from economic game theory is the competitive advantage gained by the first company to enter a certain market [63]. Our interaction design allows Vehicle $J$ such an advantage by limiting Vehicle $M$’s response in return, i.e. $M$ can only $\mathrm{punish}$ a forced join but not $\mathrm{block}$ it. When *deceiving* Vehicle $M$ into inaction by not advertising its intent, Vehicle $J$ secures its *first mover advantage.* The positive effect of this is evident in the *rate of improvement* Vehicle $J$’s average payoff has between $\text{Test Group I}$ and $\text{Test Group II}$ compared to that of Vehicle $M$. This rate of improvement averages just 5% for Vehicle $M$ across both Rulesets, whilst Vehicle $J$ sees a 26% improvement in turn. Thus, Vehicle $J$ secures a relative advantage. This advantage may carry important ramifications to the success of autonomous vehicles if they are able to capitalise on their inherent advantages in reaction time to secure the first move. Literature suggests that in Stackelberg Oligopoly games, an aggressive first move by the Leader Firm can often induce the rival Follower Firm to take a more ‘submissive’ action that favours the Leader Firm [64]. Of course, this also comes with its own pitfalls. By forcing the join, Vehicle $J$ is *committing* to joining ahead of $M$. This commitment can often prove costly if Vehicle $J$ is unable to follow through with it. Indeed, we observe the negative impact of the failure to maintain a commitment in our own results. The average payoff for Vehicle $J$ in $\mathrm{force/abort}$ interactions is approximately 2.6 times *worse* than the average payoff in $\mathrm{indicate/wait}$ interactions in both rulesets.            

### Effect on Interaction Safety

Given the findings in [2], we expected to see a reduction in the occurrence of crashes and near misses with communication. Our observations indeed demonstrate this trend. We see Ruleset 1’s $\text{Test Group I}$ reduce near misses by 68% compared to the $\text{Control Group}$, whilst Ruleset 2’s $\text{Test Group I}$ reduces crashes and near misses by 31% and 41%, respectively, compared to the $\text{Control Group}$. What is worthy of note is that compared to $\text{Test Group I}$, $\text{Test Group II}$ in both rulesets delivered a far greater reduction in near misses under Ruleset 1 (82%) and more than double the reduction under Ruleset 2 (70% and 87% for near misses and crashes, respectively). This suggests a profound *positive* impact on interaction safety from making $J$’s communication discretionary. This is an interesting observation, as one would expect the apparently more risk tolerant approach of choosing to force an interaction to have more dangerous consequences. We examined the data by comparing all 115 $\mathrm{block/join}$ crashes which occurred under Ruleset 2, $\text{Test Group I}$, against their mirror occurrences in $\text{Test Group II}$. Whilst none resulted in a crash, we discovered that in 87 out of the 115 interactions in $\text{Test Group II}$, Vehicle $M$ would have $\mathrm{allowed}$ Vehicle $J$ to join in retrospect. This is the Stackelberg Oligopoly phenomenon described earlier in action; Vehicle $J$’s *aggressive* first move coaxed Vehicle $M$ into more submissive behaviour, which in these 87 cases prevented a crash.

### Effect on Interaction Efficiency (Non-ideal Outcomes)

We see a clear reduction in non-ideal outcomes as more information is made available via communication, especially with the $\mathrm{block/join}$ outcome. We note the slight *increase* in $\text{allow/wait}$ outcomes in the $\text{Test Group II}$ runs compared to $\text{Test Group I}$ (+0.05% and + 0.01% under Rulesets 1 and 2, respectively), despite the over-all reduction compared to the $\text{Control Group}$. This can be explained by considering Vehicle $J$’s added ability to *change its mind* in $\text{Test Group II}$. That is, to back out of a forced join. If we exclude these interactions, the percentage of $\text{allow/wait}$ outcomes goes down to 1.25%. This is a more appropriate direct comparison since Vehicle $J$ does not have the option to change its mind in the other simulation groups.

### Notable Observations

Despite the appeal and tangible benefit of masking its intent from Vehicle $M$, Vehicle $J$ did not adopt $\text{force join}$ as a pure strategy. In fact, Vehicle $J$ *chose* to signal its intent to Vehicle $M$ in 38% of all interactions across both rulesets. This means that Vehicle $J$ *still* found an advantage in communicating its intent under the right circumstances in a significant number of cases.

Interestingly, whilst it is noted that Ruleset 2 generally performed worse than Ruleset 1, the gap between the two rulesets is significantly reduced as more communication is introduced. For example, Ruleset 2’s $\text{Control Group}$’s average utility (for both vehicles) was 45% worse than Ruleset 1’s $\text{Control Group}$. This gap is narrowed to 32% in $\text{Test Group I}$ and down to just 7.25% in $\text{Test Group II}$. This convergence in average utility suggests that intelligence on the other vehicle’s *current state* (in this case, $\mathrm{attention}$ and $\mathrm{intention}$) can go a long way in counteracting the effect of having no knowledge of the other vehicle’s kinematic attributes and preferences. This finding should be treated with caution, however, as we have not conducted enough trials to test its sensitivity to preset parameters. Nevertheless, we can observe a clear correlation between communication and improved utility. This improvement is much more pronounced when at least one vehicle can *choose* when and if to communicate.

### On Bayesian Statistics and Bounded Rationality

Whilst our model adopts Bayesian inference as the basis for vehicles’ decision-making, we acknowledge that this represents an idealised form of rationality. Bayesian inference is often used to solve games with limited information; thus, it is a form of addressing bounded rationality in and of itself. Indeed, human road users typically operate under conditions of bounded rationality, relying on heuristics, limited information, and satisficing strategies rather than precise probabilistic reasoning. Bayesian inference is ultimately a principled, mathematical approach in which humans generally do not engage—at least not on a conscious level. Nevertheless, Bayesian games do offer a transparent and principled method to represent uncertainty, capture the influence of available information on interaction outcomes, and formalise the updating of beliefs as new information comes to light. This makes Bayesian games particularly suitable as a modelling baseline, especially given its widespread adoption in autonomous driving research. Our use of Bayesian inference is thus not intended to suggest that human drivers are perfectly rational, but rather to provide a systematic and extensible foundation for comparing interaction outcomes under different communication strategies.

## Conclusion

We set out in this paper to examine whether discretionary communication can enhance the outcome of road user interaction from a game-theoretic perspective. We investigated two hypotheses. First, that vehicles which communicate selectively achieve better payoffs than those which communicate unconditionally. Second, that within a non-cooperative game-theoretic framework, communication (even when selective) can yield safer and more efficient interactions.

Our experiments reinforce our previous findings that non-cooperative game theory is a viable framework to model the exchange of communication between road users [2]. Furthermore, our introduction of discretionary communication to allow the joining vehicle to gain first mover advantage has shown promising results. Namely that the joining vehicle is able to complete more interactions in its favour, thus demonstrating that there is advantage in masking one’s own intent under the right circumstances. We also find that by behaving more ‘aggressively’, the joining vehicle elicits more ‘submissive’ behaviour from the main-lane vehicle. This creates an emergent phenomenon where interaction safety is improved as conflicts are reduced. We conclude that non-cooperative communication can produce emergent benefits in safety and efficiency for all parties involved.

We amplified the occurrence rate of explicit communication in this experiment to facilitate comparison. Thus, future work should investigate the sensitivity of our results to the occurrence rate of explicit signals. Our work would also benefit from sensitivity analysis of factors such as the crash penalty, the wait penalty factor $\omega$ and the punitive sensitivity factor ${\alpha}_{P}$. By better understanding how the different variables influence the results of the simulation, one can draw wider conclusions on how human and autonomous preferences can shape an interaction. Real-world validation of parameters would also aid in supporting the assumptions of this model and expanding its application in real-world settings.    

In this paper, we only explored one aspect of deception: masking intent. Future iterations on the model should investigate other forms of deception, such as giving misleading information, exaggeration of vehicle capabilities or feigning inattention to discourage an opponent from action. With that said, there is a clear conceptual distinction between simply *exercising discretion* with what to communicate and *actively communicating misleading information*. This is particularly important in the context of autonomous vehicles. Unlike human drivers, autonomous vehicles are expected to conform to strict safety and transparency standards, which makes the intentional use of deception problematic from both regulatory and societal trust perspectives. Thus, although our model allows for the exploration of deceptive signalling as a theoretical construct, we emphasise that its application to autonomous vehicles must be framed within clear ethical boundaries and accountability structures.

## Data Availability

The model source code and the generated data used to support the findings of this paper are available from the University of Leeds at 10.5518/1608.
